# Modulating Fundamental Resonance in Capacitive Coupled Asymmetric Terahertz Metamaterials

**DOI:** 10.1038/s41598-018-34942-2

**Published:** 2018-11-13

**Authors:** S. Jagan Mohan Rao, Yogesh Kumar Srivastava, Gagan Kumar, Dibakar Roy Chowdhury

**Affiliations:** 1 Department of Physics, Indian Institute of Technology Guwahati, Guwahati, 781039 Assam India; 2 Division of Physics and Applied Physics, School of Physical and Mathematical Sciences, Nanyang Technological University, Singapore, 637371 Singapore; 3 Mahindra Ecole Centrale, Jeedimetla, Hyderabad, 500043 Telengana India

## Abstract

In this work, we experimentally investigate near-field capacitive coupling between a pair of single-gap split ring resonators (SRRs) in a terahertz metamaterial. The unit cell of our design comprises of two coupled SRRs with the split gaps facing each other. The coupling between two SRRs is examined by changing the gap of one resonator with respect to the other for several inter resonator separations. When split gap size of one resonator is increased for a fixed inter-resonator distance, we observe a split in the fundamental resonance mode. This split ultimately results in the excitation of narrow band low frequency resonance mode along with a higher frequency mode which gets blue shifted when the split gap increases. We attribute resonance split to the excitation of symmetric and asymmetric modes due to strong capacitive or electric interaction between the near-field coupled resonators, however blue shift of the higher frequency mode occurs mainly due to the reduced capacitance. The ability of near-field capacitive coupled terahertz metamaterials to excite split resonances could be significant in the construction of modulator and sensing devices beside other potential applications for terahertz domain.

## Introduction

In the electromagnetic spectrum, terahertz gap exists between microwave and infrared regions and is potentially significant to a variety of applications ranging from medical sciences to engineering^1^. Many natural materials inherently do not respond to terahertz radiation. However in this scenario, metamaterials are quite promising to make devices such as antennas, modulators, next generation high speed communication devices, ultra-sensitive detectors, sensors etc. for terahertz frequencies^1–11^. Metamaterials exhibit many interesting properties such as negative refractive index^12^, perfect focusing^13^, cloaking^14,15^, and resonance modulation in the active and passive modes^16–26^ etc. Fundamentally Metamaterial (MM) is an arrangement of artificial structured elements designed to achieve unusual but desired electromagnetic (EM) properties^27–33^. Typically, split ring resonators (SRRs) are the basic building blocks of metamaterials with lattice constant much smaller than the excitation wavelengths^34–39^. In recent times, a lot of emphasis has been given to the fabrication of terahertz metamaterials and realization of terahertz photonic devices. Because of the longer wavelengths of terahertz radiation, it is relatively convenient to fabricate metamaterials at terahertz frequencies. In order to get suitable spectral response from the metamaterial structures, numerous approaches based on metamaterials have been employed which include modification of substrate parameters^40,41^, lumped capacitors or varactors based MM^36^, usage of liquid crystals^42,43^, ferromagnetic and ferroelectric techniques^42^, microelectromechanical systems (MEMS) based switches^44^, resonance modulation based on near-field interactions between nearest neighbor SRRs^45–52^ etc. In our present study, we have focused on near-field manipulations between nearest neighbor SRRs. Metamaterials in near-field coupled configuration can consist of more than one SRR (meta-atoms) in a unit cell and intelligently manipulating the near-field coupling between these resonators, can have significant impact on the metamaterials responses. Therefore, controlling the near-field coupling in metamaterials is extremely crucial in order to determine its role in designing and fabrication of terahertz photonic components viz. modulators, filters, polarization rotation etc. By nature, near-field coupling in metamaterials could be resistive, capacitive or inductive. Several near-field coupling mechanisms have been studied in recent past using different schemes, however these coupling mechanisms have been extensively electric and magnetic in nature^19,20,53,54^. In our earlier works, we have investigated broadside coupling along with near-filed inductive coupling in planar terahertz metamaterial^55^.

In this paper, we have focused mainly on the capacitive near-field coupling in a terahertz metamaterial. Although, the investigations on near-field coupling in terahertz metamaterials have led to many interesting physics and engineering, but actualization of terahertz devices using such coupling mechanisms require other challenges to be addressed. This includes engineering the near-field capacitive coupling to excite sharp low bandwidth resonance which is not demonstrated earlier to the best of our knowledge. In this context, gap to gap near-field capacitive coupling between adjacent SRRs can result in either blue shift or red shift of the fundamental resonance, based upon the relative positions of the split gap of SRRs^56^. This provides an extra degree of freedom to manipulate and control the phase and amplitude of the terahertz wave propagation. Further, near-field interaction between the adjacent SRRs results in a strong redistribution of energy in comparison to the single SRR giving an additional degree of freedom in designing metamaterials. In this paper, we specifically explore the asymmetric gap to gap near-field capacitive coupling between a pair of SRRs in a coupled unit cell. This is achieved by changing the gap size of one resonator with respect to the other. We have extended our study from strong near-field regime to weak near-field regime. Because of the interactions between the asymmetric split gaps, the asymmetric and symmetric coupled resonance modes are excited which results in low bandwidth and frequency tunable resonance modes.

## Results

### Metamaterial design and experimental details

**Figure 1:**
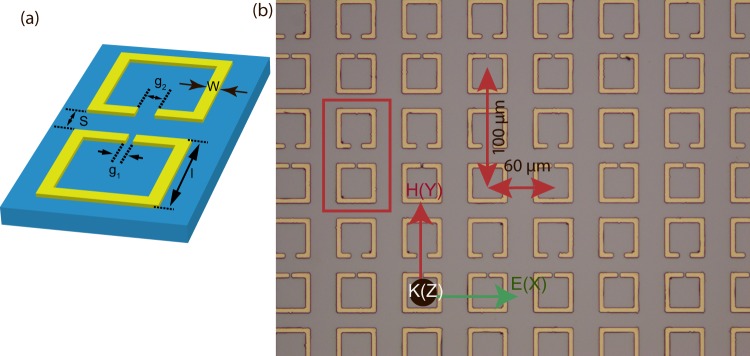
(**a**) Schematic of the unit cell comprising of two SRRs in a coupled THz metamaterials. The blue regions indicate substrate while the yellow regions represent metallic areas. Each of the SRRs has an outer dimension of *l* × *l* = 36 × 36 *μm* and gap (*g*_1_) of bottom SRR is 2 *μm*, gap of top SRR is *g*_2_ (*g*_2_ = 2, 6, 10…28 *μm*), width (w) of SRR is 4 *μm*. ‘S’ represents the separation between two SRRs in Y-direction (S = 2, 6, 10 and 14 *μm*). (**b**) Optical microscope image of fabricated sample.

The design of terahertz (THz) metamaterials is crucial in accomplishing the desired electromagnetic response. In our proposed metamaterial configuration, metamolecule (unit cell) is comprised of two split ring resonators (SRRs) with gaps facing each other. A schematic of the proposed configuration is shown in Fig. 1(a). We have assumed silicon as the substrate. The unit cell is chosen to be sufficiently bigger in size (*P*_*x*_ = 60 *μm* and *P*_*y*_ = 100 *μm*) compared to the metamolecule or SRR pair in order to avoid any undesired near-field couplings between the neighboring unit cells. In such a metamolecule design, both the SRRs i.e. top and bottom as depicted in the figure are 36 × 36 *μm* in length and breadth with an aluminum layer of thickness of 200 *nm* deposited at the top side. The capacitive gap and line width of the bottom resonators are assumed to be *g*_1_ = 2 *μm* and *w* = 4 *μm*, respectively. The above mentioned parameters remain constant throughout the analysis for the bottom resonator. However, in case of top resonator, the line width is same i.e. *w* = 4 *μm*, but the top capacitive gap (*g*_2_) is varied from 2 *μm* to 28 *μm*. The inter resonator separation (S) is varied from 2 *μm* to 14 *μm* in the step size of 4 *μm*.

For the fabrication of samples, we used conventional photolithography technique in the clean room environment. The samples were fabricated on a 500 *μm* thick high resistivity (>5000 ohm-cm) silicon substrate. Followed by photolithography, a 200 *nm* thick aluminum was deposited by using a thermal evaporator, after which a lift-off process enabled the formation of the SRR array. Optical microscope images of the fabricated samples are shown in Fig. 1(b) with the detailed geometric dimensions. The measurements were carried out using a typical 8f confocal terahertz time-domain spectroscopy (THz-TDS) system consisting of a photoconductive antenna transmitter and a receiver. The metallic antenna on GaAs chip is dc biased at 70 V and excited by 130 fs optical pulses with a wavelength of 800 *nm* and a repetition rate of 80 MHz from a Ti: sapphire oscillator laser system generating THz pulses with a bandwidth of 0.2–3 THz. The polarization of the incident THz electric field is aligned parallel to the gap bearing arms of the SRRs in order to excite the fundamental LC resonance mode (Fig. 1(b)) based on the experiments, THz signal was measured in the time domain after transmitting through the metamaterial samples. It was Fourier transformed to obtain the frequency domain spectra, which is further normalized with the signal from a bare silicon substrate of the same thickness (as reference) as used in the metamaterials samples. All the measurements were done at room temperature and in a dry nitrogen atmosphere in order to mitigate the effect due to water vapor absorption. We have studied four different sets of samples corresponding to four different separations (S) between SRRs with a fixed bottom gap (*g*_1_), while the top resonator gap (*g*_2_) is changed gradually as indicated in Fig. 1(a). The separation between the resonators is symbolized by S in Y direction. In this study, different values of S are considered to be as 2, 6, 10 and 14 *μm*. We have examined the response of THz transmission through the proposed design for various capacitive gaps (*g*_2_ = 2, 6, 10, 14, 18, 22, 26 and 28 *μm*) of the top resonator for four different separations (S) both experimentally and numerically. For our numerical study, commercially available numerical software, CST Microwave Studio is employed and tetrahedral meshing is adopted for simulating the metamaterials geometry. The boundary conditions are taken as periodic in the full wave numerical simulations. We have used waveguide ports as the source and detector. The results of the THz transmission through the planar THz metamaterial systems are discussed elaborately in the next section.

## Discussions

**Figure 2:**
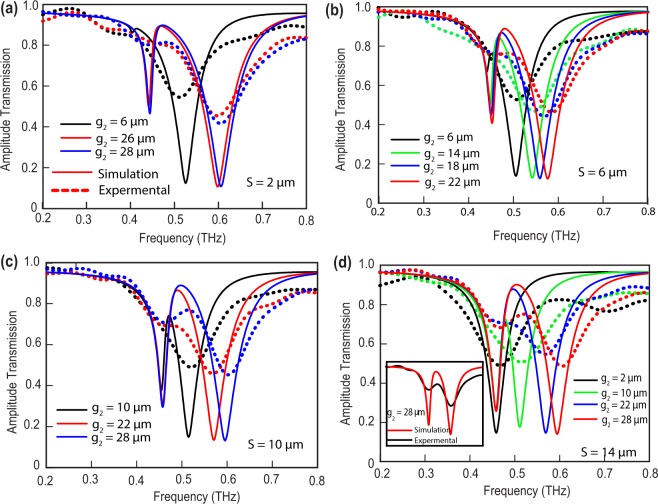
Experimental and numerically calculated amplitude spectra for four different separations. Thick lines represent the simulation results and the dotted lines represents transmission data. *g*_2_ = 28 *μm* case is enlarged in inset of (**d**) for elaboration.

**Figure 3:**
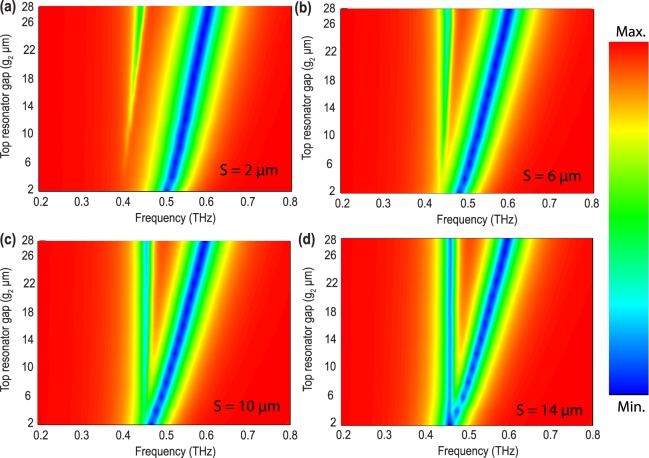
Contour plot of numerically simulated THz transmittance for four different separation between two SRRs. Color bar shows the magnitude of transmission intensity. (**a**) Represents contour plots for separations between two SRRs i.e. *S* = 2 *μm* case. (**b**–**d**) represents contour plots for *S* = 6, 10, 14 *μm*, respectively.

The THz transmission results through the fabricated metamaterial samples for four different separations (S) are shown in Fig. 2. In Fig. 2(a), the separation (S) between top and bottom SRRs is fixed at 2 *μm* and the top SRR gap (*g*_2_) is varied for 6 *μm*, 26 *μm* and 28 *μm*. In case of *g*_2_ = 6 *μm*, high frequency resonance mode is observed at around 0.52 THz and low frequency mode at 0.41 THz. As the top resonator gap changes to 28 *μm*, we observed a blue shift in the higher order mode i.e. frequency shifts to 0.6 THz while the low frequency resonance remains almost unperturbed. In case of 6 *μm* separation, high frequency mode appears at 0.5 THz and the low frequency mode appears around 0.44 THz. As the top gap (*g*_2_) increases, the high frequency mode goes through blue shifting just like the previous case. Similar trends are observed for separations 10 *μm* and 14 *μm*. We have performed experiments to validate our results which are plotted in dashed lines in Fig. 2 along with the numerical simulations. Note that the resonance frequencies in the experiments follow simulations well however the linewidths are not in good match. The difference in the line-width of the resonance is primarily due to low spectral resolution of measurement, which is limited by the Fabry-Perot reflection pulse from the rear surface of the substrate. In order to develop a comprehensive understanding of near-field coupling in the proposed metamaterials, we simulated the coupled structures for several top resonator gap for all the four separations. The results are shown in Fig. 3 using contour plots. In Fig. 3, X axis is taken as frequency (THz), top resonator gap (*g*_2_) is plotted in Y axis and the color bar represents magnitude of transmission. Figure 3(a) shows the terahertz transmission for the case of separation, S = 2 *μm*, whereas, Fig. 3(b–d) shows the transmission results when the separations between the coupled SRRs are S = 6, 10 and 14 *μm*, respectively. These plots clearly indicates a resonance split when gap *g*_2_ no longer equal to *g*_1_. As *g*_2_ increases, the split becomes prominent. We attribute the split in resonance to the strong coupling between the resonators primarily. The higher mode suffers clear blue shifting and this is due to the reduced capacitance of the top resonator where *g*_2_ is increasing leading to reduction split gap capacitance of the top resonator.

**Figure 4:**
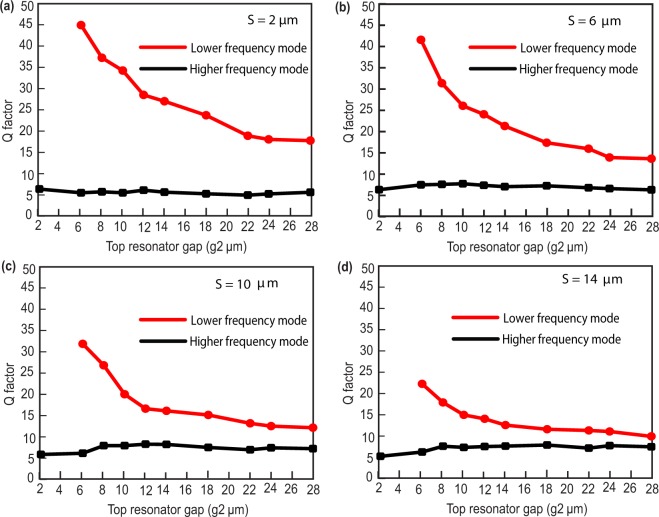
(**a**–**d**) Represents Q factor verses top resonator gap plots for S = 2, 6, 10 and 14 *μm* cases respectively.

**Figure 5:**
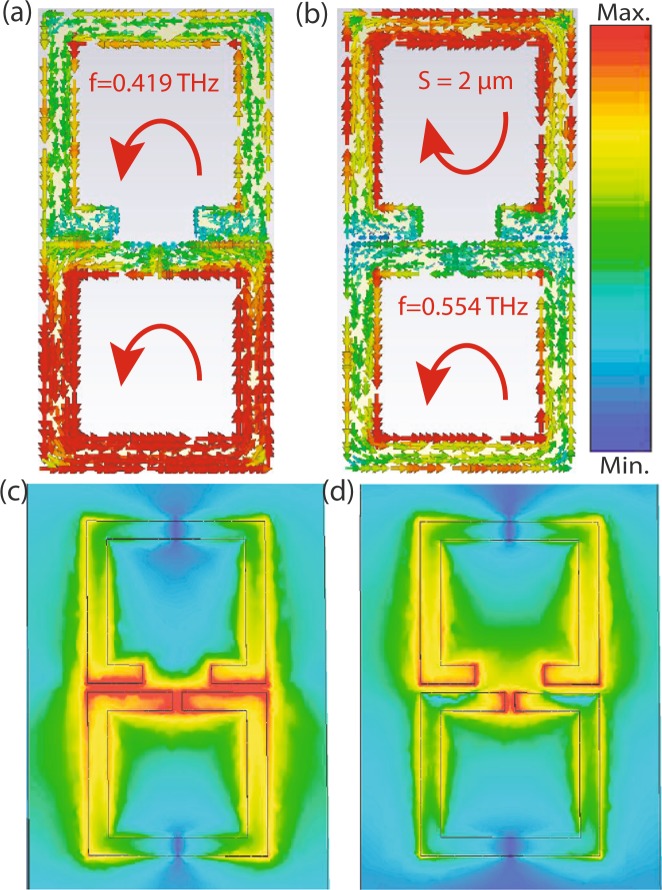
(**a** and **b**) Represent the surface current profile of S = 2 *μm* case for *g*_1_ = 2 *μm*, *g*_2_ = 14 *μm* at f = 0.419 THz and f = 0.554 THz. (**c** and **d**) Represent the electric field profiles for the same cases.

**Figure 6:**
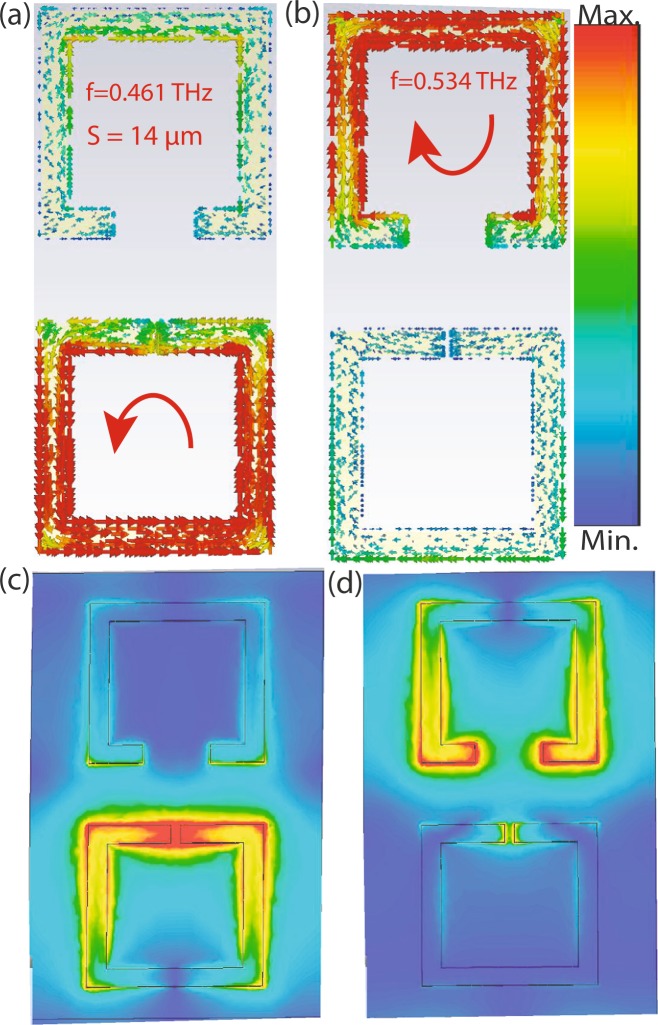
(**a** and **b**) Represent the surface current profile of S = 14 *μm* case for *g*_1_ = 2 *μm*, *g*_2_ = 14 *μm* at f = 0.461 THz and f = 0.534 THz. (**c** and **d**) Represent the electric field profiles for the same cases.

In order to understand the device performance further, we have calculated the quality factor for both the resonance modes. Quality factor which is typically defined as

1 $$Q=\frac{{f}_{r}}{{\rm{\Delta }}f}$$

(the ratio between the resonance frequency *f*_*r*_ and the bandwidth Δ*f* determined at the full width at $\frac{1}{\sqrt{2}}$ maxima in case of amplitude transmission). We have calculated quality factor for all four different separations and for each separation, we have considered top resonator gap *g*_2_ = 2, 6, 8, 10, 12, 14, 18, 22, 24 and 28 *μm*. To evaluate quality factor, first we have calculated the full width of each resonance mode at $\frac{1}{\sqrt{2}}$ maxima. For S = 2 *μm* case, when *g*_1_ = *g*_2_ = 2 *μm*, we have only one resonance mode at 0.5024 THz with full width 0.08 THz and the corresponding Q factor is 6.28 and for *g*_2_ = 6 *μm* case, we have two resonance peaks, first resonance peak at 0.405 THz with Δ*f* = 0.009 THz and corresponding Q factor is ≈45. The second resonance peak appears at 0.524 THz with Δ*f* = 0.1 THz and corresponding Q factor turns out to be 5.24. Similarly, we have calculated Q factor for *g*_2_ = 10, 14, 18, 22 and 28 *μm* and results are shown in Fig. 4(a). For S = 6, 10 and 14 *μm* also, the calculated Q factors are shown in Fig. 4(b–d), respectively. In all the cases, we observed lower frequency mode has higher Q factor compared to the higher frequency mode. In case of minimum separation (S = 2 *μm*) the Q factor observed is maximum. In order to validate our physical explanations, we have monitored the induced surface current profiles and electric field profiles for strongly coupled regime (Fig. 5). In case of S = 2 *μm* separation with asymmetric split gaps in the resonators, surface currents are shown corresponding to lower and higher resonance modes, see Fig. 5(a,b). The induced surface currents in the resonators are in phase in case of lower resonance mode and 180° out of phase in case of higher resonance mode. This clearly indicates that the resonators are strongly coupled through electric field lines or in other words the resonators are capacitively coupled through the split gaps. In order to validate this explanation, we have further simulated the electric field profiles at the split resonance dips as shown in Fig. 5(c,d). Electric field profiles clearly indicate that the resonators are strongly coupled at the split resonances. This also explains the trend in Q factor along with resonance frequency shift. S = 2 *μm* (Fig. 4a) signifying strongly coupled regime, the resonators are strongest coupled therefore leading to maximum deviation in Q factor along with maximum shift in frequency between the resonance dips (Figs 2(a) and 3(a)). However as we move to weakly coupled regime, for example S = 14 *μm*, the difference in Q factor between the modes are lesser. Because of weak capacitive interactions between the resonators the resonance dips are also not deviated from their intrinsic or uncoupled positions. In order to validate this point, we have further simulated the surface current distributions and electric field distributions of the resonators at weakly coupled regime (S = 14 *μm*) (Fig. 6). One may note that the individual resonators are strongly excited at the resonance dips signifying the coupling as weak or almost absent. At the low frequency mode, the lower resonator is excited to its fundamental mode (Fig. 6(a)). Similarly at the higher frequency mode the top resonator is excited to its fundamental mode (Fig. 6(b)). It clearly shows that the resonators are very weakly coupled and therefore resonating close to their intrinsic resonance frequencies.

## Conclusions

In this work, we have examined the ability to tune resonance behavior via near field capacitive coupling in planar terahertz metamaterials. This has been achieved by manipulating the near field electric interactions via changing one resonator split gap with respect to the other resonator split gap for several inter resonator separations. Introducing asymmetry by changing the split gap in one resonator with respect to the other resonator, results in the split in the fundamental resonance mode when operated in the strong near field coupled regime. The split occurs because of the strong near field capacitive/electric interactions between the resonators. We have further calculated Q factor for the lower and higher resonance modes for different inter resonator separations. We observed that the lower resonance mode has significantly higher Q factor compared to the higher frequency resonance mode. We believe that the higher Q factor observed in the lower frequency resonance mode is due to the strong electric coupling between the resonators which results in larger effective inductance. In near field interaction of the resonators, we also observed a blue shift in the higher frequency split resonance. This is attributed to the enhanced split gap size leading to reduced capacitance value. The modulation of resonances in capacitive coupled planar terahertz metamaterial systems has great potential in manipulating and controlling electromagnetic waves which can ultimately result in novel applications for terahertz frequency domain such as designing sensors, antennas, modulators, switches etc.

## Methods

We have fabricted MM samples using conventional photolithography technique in the clean room environment and characterized using typical 8f confocal terahertz time-domain spectroscopy (THz-TDS) system consisting of a photoconductive antenna transmitter and a receiver. We have used finite element frequency domain solver for the simulations in CST Microwave Studio package.
